# PTSD and the War of Words

**DOI:** 10.1177/2470547018767387

**Published:** 2018-04-16

**Authors:** Adam M. Chekroud, Hieronimus Loho, Martin Paulus, John H. Krystal

**Affiliations:** 1 Department of Psychiatry, Yale University, New Haven, CT, USA; 2 Data Science Division, Spring Health, New York City, NY, USA; 3 Laureate Institute for Brain Research, Tulsa, OK, USA; 4 US Department of Veterans Affairs National Center for PTSD, VA Connecticut Healthcare System, Newington, CT, USA

**Keywords:** PTSD, history of medicine, disease names, psychiatry, culturonomics, media, *New York Times*

## Abstract

Trauma-related symptoms among veterans of military engagement have been documented at least since the time of the ancient Greeks.^1^ Since the third edition of the Diagnostic and Statistical Manual in 1980, this condition has been known as posttraumatic stress disorder, but the name has changed repeatedly over the past century, including shell shock, war neurosis, and soldier’s heart. Using over 14 million articles in the digital archives of the *New York Times*, *Associated Press*, and *Reuters*, we quantify historical changes in trauma-related terminology over the past century. These data suggest that posttraumatic stress disorder has historically peaked in public awareness after the end of US military engagements, but denoted by a different name each time—a phenomenon that could impede clinical and scientific progress.

## Introduction

Trauma-related symptoms among veterans of military engagement have been documented at least since the time of the ancient Greeks,^1^ with modern names including *shell shock*, *battle fatigue*, and* Post Traumatic Stress Disorder* (PTSD).^2,3^ Historically, the experiences of veterans have played a major role in shaping our understanding of trauma-related disorders.^4^ In parallel, clinicians and researchers continue to debate definitions and diagnostic classifications of trauma-related disorders,^5,6^ which have important implications for patients in terms of diagnosis, treatment, insurance, disability status, and forensics.

Mainstream news media offer another lens through which we can understand how this debate and accompanying renaming process has unfolded over time. Therefore, to advance the debate, we set out to quantify historical changes in trauma-related terminology over the past century from the perspective of mainstream media.

## Methods

We examined word use in the mainstream media, as reflected by articles in the digital archives of the* New York Times*, *Associated Press*, and *Reuters*. Using the New York Times Developer Application Programming Interface, we queried 14,138,283 articles published from 1900 to 2016 for key words in the title, byline, or body of the article. Our search criteria were as follows: articles had to contain at least one military association word (“veteran,” “soldier,” “military,” or “armed forces”), as well as a PTSD moniker of interest (selected manually by the authors). To account for the fact that more articles are published per year now than in 1900, we focused on the percentage of all articles each year that included each search term, rather than the absolute number of articles with that term. All code (github.com/HLoho/NYT-PTSD) and data (developer.nytimes.com/) from this study are available online. Note that 27 trauma-related terms were queried in total, but only four of the most common are presented in these analyses (see Supplementary Material for a full list of terms).

## Results

**Figure 1.**
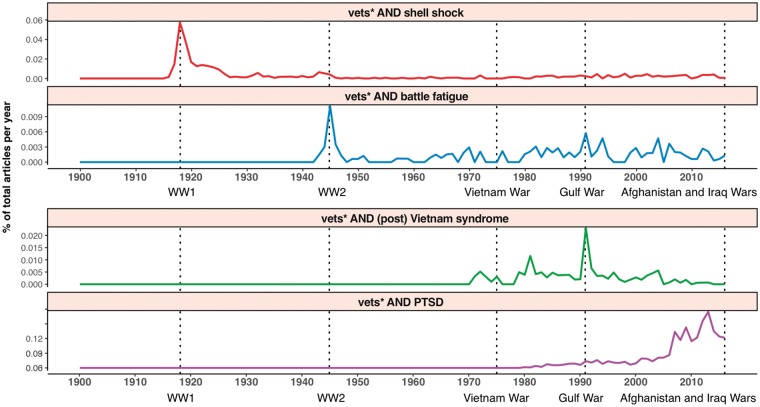
The ebb and flow of veterans and trauma-related syndrome mentions in mainstream media. Annual percentage of articles mentioning veterans and specific terms for PTSD. Source: Reproduced with permission from *New York Times*, *Reuters*, and *Associated Press*, 1900–2016. Vets*: “veteran or soldier or Military or armed forces.”

Figure 1 illustrates the ebb and flow of trauma-related syndrome mentions in mainstream media: around the end of each major war (indicated by dotted vertical lines), a new term for PTSD comes into public consciousness and replaces its predecessor. *Shell shock* gave way to *battle fatigue*, which gave way to *Post Vietnam Syndrome*, which was finally overtaken by *PTSD*. The rate at which terms then fade into obscurity in the post-war years perhaps illustrates how quickly the mental health sequelae of war are forgotten in mainstream media.

**Figure 2.**
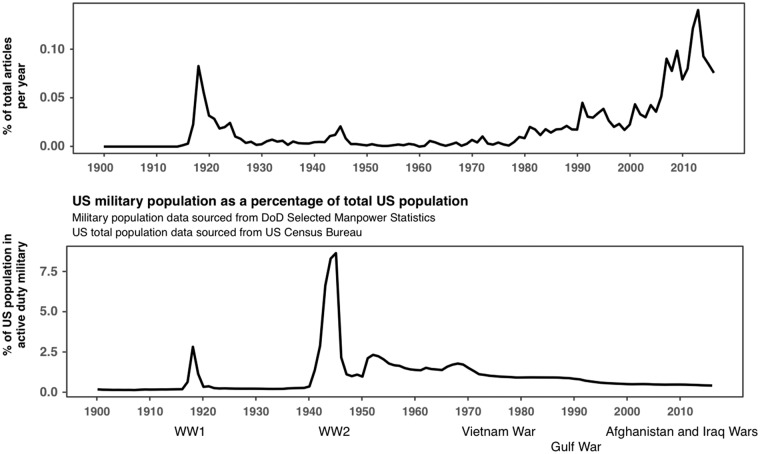
Media discussion of veterans and PTSD* does not simply track US Military population. Overall mentions of veterans and term for PTSD. Source: Reproduced with permission from *New York Times*, *Reuters*, and *Associated Press*, 1900–2016. Vets*: “veteran or soldier or Military or armed forces”; PTSD*: any from Figure 1.

A simple factor that might influence this phenomenon is the percentage of US population in active military duty. With this in mind, Figure 2 shows the percentage of articles each year that included a veteran term and a PTSD moniker (upper panel), alongside the percentage of the US population currently on active duty in the military (lower panel).^7–11^After the Vietnam War, the percentage of PTSD mentions has broadly increased while the number of active duty personnel decreased (and there is no significant correlation between the two time-series overall, *r*(115) = −0.078,* p* = 0.40). This broad trend toward greater discussion of PTSD may indeed reflect an underlying trend toward increased discussion of mental illness since the 1950s,^12,13^ and may be explained by a generational change in the willingness of veterans to talk about their experiences and of the public to listen to them.

## Discussion

Language changes as understanding changes. *PTSD* symptoms were termed *shell shock* in the First World War because it was thought they were caused by concussive physical trauma due to shells used in trench warfare.^2^ This in turn informed the design of early experimental studies that showed, for example, that veterans with *shell shock* exhibited greater increases in heart and respiratory rate than healthy controls when exposed to gunfire or sulfuric flame in laboratory settings^14^ (for a review, see Krystal et al.^15^). During the Second World War, studies of *irritable heart* or *soldier’s heart* focused on exaggerated arousal responses and sympathetic nervous system activity (e.g., Crile, 1940).^16^ After the Second World War, the Diagnostic and Statistical Manual (DSM)-I introduced *gross stress reaction* in 1952, but omitted the term in the second edition in 1968 during the Vietnam War.^2^ It is likely that authors of the DSM II never experienced treating *battle fatigue* and PTSD-like symptoms during the First World War and the Korean War, and respected psychiatrists working in Vietnam felt that the mental health issues they encountered were encompassed by other diagnoses.^17^ However, with publication of the DSM-III in 1980, the idea of trauma-related neurosis came back. The introduction of *PTSD* broadened the phenomena that could account for PTSD (including non–war-related factors, e.g., sexual abuses), and broadened the phenomenology of the condition to include reexperiencing, numbness/depression, hyperarousal, and cognitive symptoms. Accumulated over decades of research, our current knowledge about *PTSD* now recruits genetic, epigenetic, and neuroscientific methods to understand fear conditioning, dysregulated circuitry, and memory reconsolidation.^18,19^

Along with updates in scientific knowledge, changing cultural and societal factors also influenced the changing nomenclature of post-war syndromes.^3,4^ A clear example of this occurred in 1922, when the British government’s War Office Committee of Enquiry into “Shell Shock” officially declared that *shell shock* was not a valid battle casualty and recommended the term be banned.^20^ Perhaps the British Government and its citizens wanted to forget and move past the pain of the war, either consciously or subconsciously; regardless, whatever social factors were in play changed the nomenclature of post-war syndromes and pushed for *shell shock* to be forgotten. Of course, this does not mean that the renaming of post-war syndromes was deliberate. The evolving nomenclature represents the haphazard attempts of the medical community to make sense of the broad range of war-related physical and psychological symptoms that afflict veterans returning home from combat. These symptoms were historically poorly understood and thus poorly classified.^21^ That is why, for example, the mental and physical symptoms experienced by long-term prisoners of war in the Second World War were never formally recognized or named by the medical community.^22^ That is also why terms such as *Vietnam syndrome* and *battle fatigue* may represent more than just the symptoms of what we now call *PTSD*. But even to this day, the construct of PTSD itself is imperfect and based on a consensus of symptoms, a consensus that arguably does not always move the field forward.^23^ Thus, this messy evolution of disease names occurred in the context of both scientific progress and societal evolution.

In light of this history of shifting names, we must be careful to avoid losing the accumulated expertise and awareness that led to those gains in knowledge. *PTSD* has a history of particularly disjointed research. Promising physiological findings from the First World War and Second World War physicians were abandoned in favor of psychodynamic and behavioral approaches, only to be resumed years later in the 1960s.^15^ This pause in neurobiological research had clear consequences: placebo-controlled clinical trials of pharmacological PTSD treatments lagged behind other mental illnesses such as depression and schizophrenia.^15^

The renaming process could also be detrimental to improving awareness of PTSD, potentially reducing treatment rates and increasing the overall burden of PTSD. Over time, naming iterations may contribute to lapses in public awareness, making it too easy to forget that veterans have likely suffered from PTSD for as long as veterans have suffered from war. This lack of awareness could make it easier for governments to hide the magnitude of the problem in the aftermath of military engagements, as occurred when the British government banned the term *shell shock*.^20^ Changing the name of a disease or censoring its mention entirely could allow the public to forget the human cost of war.

Future investigations incorporating the rich perspective of mainstream media publications can help to unpack the complex relationship between this renaming process and stigma. For example, it may help us to understand the process by which clinical definitions become more well-known and either attract or reduce stigma, eventually leading patients to embrace or hide their illness. If patients avoid associating with terms like *battle fatigue*, the terms themselves may lose their specific meaning over time. Other popular forms of media may offer another viewpoint, such as war-related movies that appear some years after military engagements (e.g., *Apocalypse Now*, *Platoon*, *Hamburger Hill*, *Full Metal Jacket*). A multifaceted approach, including analyses of semantic content of these texts, could eventually help understand self-stigma relating to PTSD, and what interventions can minimize it.^24^

## Conclusion

The way that we name and treat disease has developed tremendously over the past 100 years. With advances in digitized media archives and computational linguistic analyses, the same can now happen for how we talk about disease.^24^ These data for the first time quantify the temporal dynamics—the ebb and flow—of PTSD-related terminology in the public eye, through the lens of mainstream news media since the turn of the 20th century. Historically, discussion of veterans and *PTSD* has peaked following US military engagements but gone by a different name each time. This phenomenon of cultural forgetting can result in the discontinuity of collective knowledge from generation to generation and risks impeding the scientific community from reaching a deeper understanding of the disease. Quantifying this phenomenon can help to contextualize ongoing debate around the name of the disorder.

## Supplemental Material

Supplemental material for PTSD and the War of Words Click here for additional data file. Supplemental material for PTSD and the War of Words by Adam M. Chekroud, Hieronimus Loho, Martin Paulus and John H. Krystal in Chronic Stress
