# Supplementary material for: PTSD and the War of Words
Source: Chronic Stress (Thousand Oaks). 2018 Apr 16;2:2470547018767387. doi: 10.1177/2470547018767387 (PMC7219886; doi:10.1177/2470547018767387)
Supplement: Supplemental material for PTSD and the War of Words [file Supplemental_material.pdf]

PTSD and the war of words supplement

Term	Total Hits
(vets*) AND PTSD	3536
(vets*) AND shell shock	715
(vets*) AND battle fatigue	260
(vets*) AND neurasthenia	175
(vets*) AND (post) Vietnam syndrome	142
(vets*) AND soldier's heart	65
(vets*) AND war_neurosis	31
(vets*) AND anxiety neurosis	12
(vets*) AND battle dream	6
(vets*) AND camp disease	4
(vets*) AND effort syndrome	4
(vets*) AND traumatic neurosis	3
(vets*) AND battle exhaustion	2
(vets*) AND combat stress reaction	2
(vets*) AND psychasthenia	2
(vets*) AND barbed-wire disease	1
(vets*) AND functional nervous disorder	1
(vets*) AND lack of moral fibre	1
(vets*) AND barbed-wire psychosis	0
(vets*) AND bomb neurosis	0
(vets*) AND Disordered_Action_of_Heart	0
(vets*) AND gross stress reaction	0
(vets*) AND hyperemotivity	0
(vets*) AND perpetual bogey	0
(vets*) AND railway spine	0
(vets*) AND vegetative neurosis	0

Figure 1. NYT Terms Queried from 1900 to 2016, sorted by total hits. (Vets*) is an abbreviation for (veteran OR soldier OR military OR "armed forces"). The four terms examined and shown in the main article (PTSD, shell shock, battle fatigue, and Post Vietnam syndrome) are highlighted in yellow near the top. Other trauma monikers like neurasthenia also had a high number of total hits, but were not presented in the paper. The percentage of article mentions per year from 1900 to 2016 for “neurasthenia” and “soldier’s heart” are shown in the next two figures.

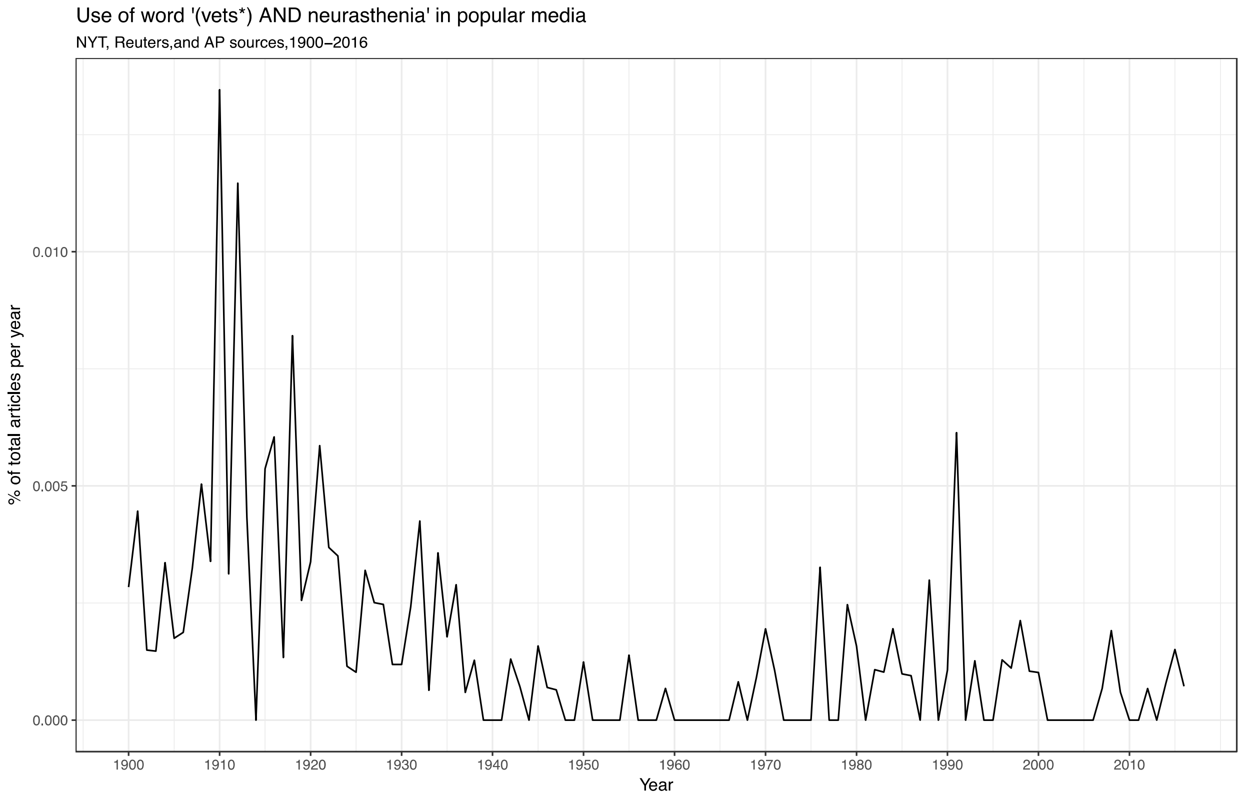

Figure 2. The yearly percentage of articles with the combined terms “(vets*)” AND “neurasthenia” are shown.

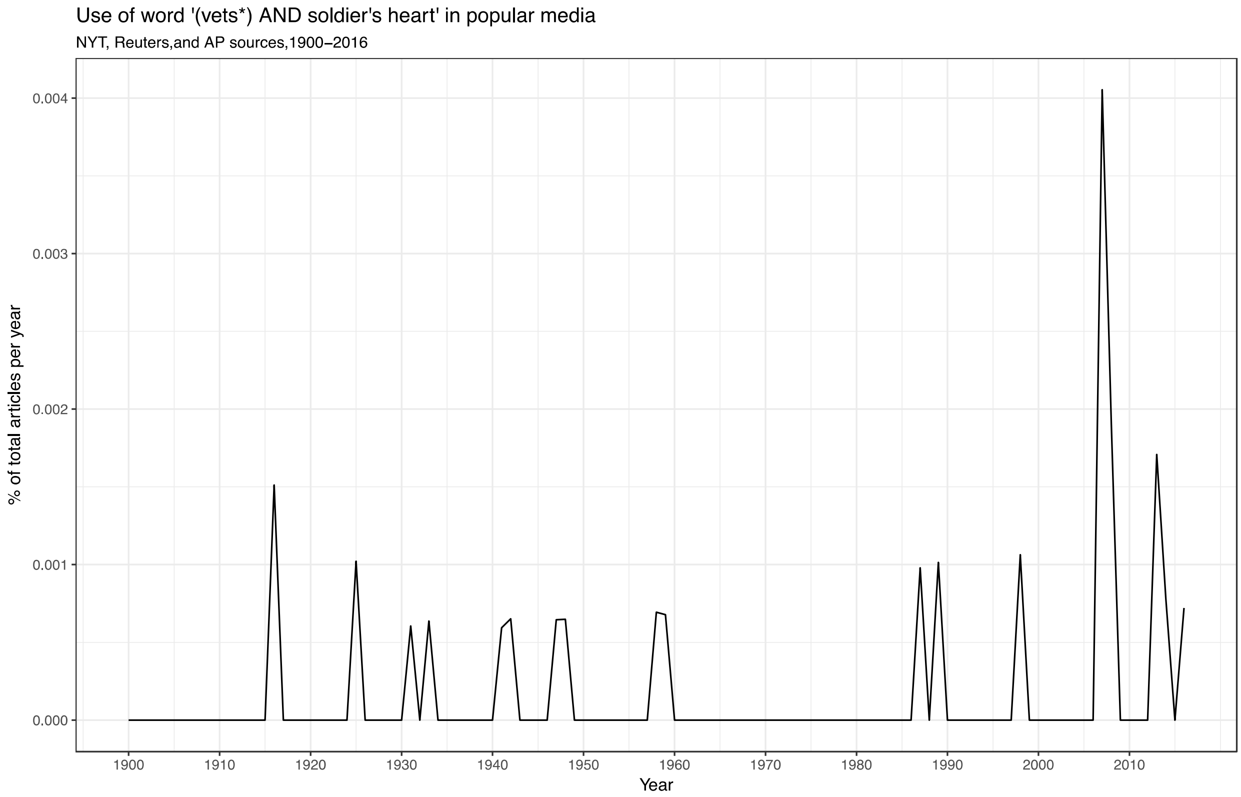

Figure 3. The yearly percentage of articles with the combined terms "(vets*)" AND "soldier's heart" are shown.
